# A New Look at Novel Cardiovascular Risk Biomarkers: The Role of Atherogenic Lipoproteins and Innovative Antidiabetic Therapies

**DOI:** 10.3390/metabo12020108

**Published:** 2022-01-24

**Authors:** Jelena Vekic, Aleksandra Zeljkovic, Khalid Al Rasadi, Mustafa Cesur, José Silva-Nunes, Anca Pantea Stoian, Manfredi Rizzo

**Affiliations:** 1 Department of Medical Biochemistry, Faculty of Pharmacy, University of Belgrade, 11000 Belgrade, Serbia; jelena.vekic@pharmacy.bg.ac.rs (J.V.); aleksandra.zeljkovic@pharmacy.bg.ac.rs (A.Z.); 2 Medical Research Center, Sultan Qaboos University, Muscat 123, Oman; k.alrasadi@gmail.com; 3 Clinic of Endocrinology, Ankara Güven Hospital, Ankara 06680, Turkey; drcesur@yahoo.com; 4 Department of Endocrinology, Diabetes and Metabolism, Centro Hospitalar Universitário de Lisboa Central, 1069-166 Lisbon, Portugal; silvanunes2004@yahoo.com; 5 Faculty of Medicine, Diabetes, Nutrition and Metabolic Diseases, Carol Davila University, 050474 Bucharest, Romania; ancastoian@yahoo.com; 6 Department of Health Promotion, Mother and Child Care, Internal Medicine and Medical Specialties, University of Palermo, 90100 Palermo, Italy

**Keywords:** lipoproteins, small dense LDL, diabetes, cardiovascular risk, GLP-1, incretins, atherosclerosis, therapy

## Abstract

The presence of residual cardiovascular disease (CVD) risk is a current dilemma in clinical practice; indeed, despite optimal management and treatment, a considerable proportion of patients still undergo major CV events. Novel lipoprotein biomarkers are suggested as possible targets for improving the outcomes of patients at higher risk for CVD, and their impact on major CV events and mortality have previously been investigated. Innovative antidiabetic therapies have recently shown a significant reduction in atherogenic lipoproteins, beyond their effects on glucose parameters; it has also been suggested that such anti-atherogenic effect may represent a valuable mechanistic explanation for the cardiovascular benefit of, at least, some of the novel antidiabetic agents, such as glucagon-like peptide-1 receptor agonists. This emphasizes the need for further research in the field in order to clearly assess the effects of innovative treatments on different novel biomarkers, including atherogenic lipoproteins, such as small dense low-density lipoprotein (LDL), lipoprotein(a) (Lp(a)) and dysfunctional high-density lipoprotein (HDL). The current article discusses the clinical importance of novel lipid biomarkers for better management of patients in order to overcome residual cardiovascular risk.

## 1. Introduction

A body of evidence from epidemiological, genetic and interventional studies strongly supports causality between low-density lipoprotein cholesterol (LDL-C) and CVD risk, emphasizing LDL-C level as a major risk factor and principal therapeutic target [1]. We are witnessing the era of innovative lipid-lowering therapies, which are able to reduce LDL-C to remarkably low levels [2]. While the newest European guidelines recommend a stepwise approach for lipid management in high-risk patients and those with established CVD, the experts also acknowledge the “the lower is, the better” concept for LDL-C levels [3]. Yet, despite optimal lipid management, a considerable proportion of CVD patients remain at risk for recurrent myocardial infarction or stroke and cardiovascular mortality [4]. Such residual risk deserves further consideration, and the importance of adherence to statins over time [5], as well as the role of nutraceuticals [6,7], has been emphasized in the recent years. Novel lipoprotein biomarkers have been suggested as possible targets for improving the outcomes of patients with CVD [8] and small dense LDL (sdLDL) seems to have the greatest potential [9,10]. 

In contrast to elevated LDL-C concentrations, there is no definitive evidence of whether low high-density lipoprotein cholesterol (HDL-C) levels should be treated [11], although this lipid abnormality has been shown as the most common form of dyslipidemia in several nationwide studies [12]. However, cholesterol content within HDL, as a single biomarker, barely reflects the diversity of the particle’s atheroprotective properties; for instance, paraoxonase-1 activity and the subclass distribution of HDL have been associated with their function and atherogenicity [13,14,15,16,17]. Indeed, further insight into HDL particles’ composition (proteome and lipidome), size heterogeneity and concentration may provide additional prognostic information [18]. Recently, HDL functionality testing has become an area of particular interest, and several emerging HDL-related biomarkers have been proposed, such as indices of cholesterol efflux, antioxidative and anti-inflammatory capacities [19]. 

The main characteristics of an ideal lipid biomarker include its ability to predict clinically significant outcomes and the patients’ response to therapy in a cost-effective manner [20]. However, unlike population-based cardiovascular prevention strategies, personalized medicine is oriented to an individual patient. In this manner, the concept of personalized laboratory medicine is based on the use of advanced technology to assist disease prevention and management [21]. The current narrative review discusses the clinical importance of novel lipid biomarkers for better management of patients in order to overcome residual cardiovascular risk.

## 2. Small, Dense LDL

Circulating LDL particles consist of several discrete subclasses, differing in size, density, composition and metabolic origin, and sdLDL is the most atherogenic fraction as a consequence of their prolonged residence in plasma, easier penetration in subendothelial space and increased susceptibility to oxidative and glycation modifications [22]. The characteristic increase in sdLDL proportion is usually associated with hypertriglyceridemia and decreased HDL-C levels, as a part of the atherogenic lipoprotein phenotype [22]. This is the most common form of dyslipidemia in metabolic syndrome and diabetic patients, which confers their increased CVD risk [23]. Smaller LDL size and/or increased proportion of sdLDL were strongly associated with CVD risk, morbidity and mortality [24,25,26]. Furthermore, sdLDL cholesterol level was the most effective predictor of a future cardiovascular event in stable coronary artery disease patients on statin therapy, as well as in high-risk patients with diabetes and hypertriglyceridemia [27].

Statins are able to favourably modulate LDL subclass distribution [28] and, more recently, promising data on improved LDL size and sdLDL reduction following 12-weeks of proprotein convertase subtilisin kexin type 9 inhibitors (PCSK9i) therapy in patients with familial hypercholesterolemia were obtained [29]. These data imply that future studies on patients with established CVD are warranted. To date, however, there is still no reliable laboratory technique for the assessment of sdLDL, which limits its wider application in clinical practice [30]. Nevertheless, several lipid indices serve as surrogate biomarkers of sdLDL, such as the increased apolipoprotein B concentrations, particularly in patients with optimal or low LDL-C levels [20], and the triglycerides/HDL-C ratio, which has a useful key role not only as a marker of insulin resistance, non-alcoholic fatty liver disease, atherogenic dyslipidemia and residual risk but also as a surrogate marker of the presence of small dense LDL [31,32]. In addition, the Lipoprint system is accessible and widely used, albeit not everywhere, for an effective lipoprotein subclass analysis, including the measurement of small dense LDL; this laboratory analysis is cheaper than other methodologies, and it has proven reliability [33].

As already mentioned, the atherogenic lipoprotein phenotype is a cluster of lipid abnormalities, including the elevation of triglyceride-rich lipoproteins [22]. As a consequence of increased production and/or impaired catabolism of triglyceride-rich lipoproteins, remnant particles are accumulated in plasma. Their presence is, in turn, directly linked to adverse modulations of LDL and HDL particles toward more atherogenic forms [34]. Mechanistically, remnant lipoproteins are enriched in cholesterol-esters and, therefore, able to further contribute to cholesterol deposition in the arterial wall. In contrast to LDL, which requires oxidative modification, remnant particles in their native form are capable of inducing foam cell formation, as well as the inflammatory response of macrophages, thus contributing to plaque vulnerability [35]. Both remnant and sdLDL cholesterol levels were strongly associated with the development of CVD in a recent study [10]. 

The results from the TNT trial clearly showed the greatest benefit of intensive statin treatment on major adverse cardiovascular events (MACE) in CVD patients with the highest remnant cholesterol levels [36]. Data from a recent real-world study showed that 4 weeks of PCSK9i therapy significantly reduced small VLDL remnants, which may be relevant for residual risk reduction [37]. So far, no definite test for the assessment of remnant particles has been established. Besides the advanced methodological approach by NMR, remnant cholesterol can be calculated from routinely lipid status parameters by subtracting LDL-C and HDL-C from total cholesterol level, and these two metrics are nearly equivalent in patients with increased triglyceride levels [38]. Alternatively, non-HDL-C, an indicator of cholesterol content in all atherogenic lipoproteins, can be used as a surrogate marker of residual risk inherent to remnant particles in patients with diabetes and hypertriglyceridemia as recommended by the actual guidelines [39]. 

## 3. Lipoprotein(a)

Another important aspect of residual risk is elevated Lp(a). The structure of this lipoprotein particle resembles LDL, with the addition of apo(a), a polymorphic apolipoprotein with several isoforms of different sizes [40,41]. Furthermore, apo(a) has a similar structure to plasminogen, although it lacks proteolytic activity. As a result, Lp(a) competes with plasminogen for fibrin binding, which prevents fibrinolysis and increases the risk of thrombus formation [41]. Additional pro-atherogenic properties of Lp(a) include its ability to pass in the subendothelial space, adhesive properties and pro-inflammatory potential [41]. The results of the AIM-HIGH study in patients with the stable coronary disease showed that those with increased Lp(a) had higher MACE risk than the patients with lower Lp(a) and comparable LDL-C levels [42]. 

As reported by the investigators of the ODYSSEY Outcomes trial, the greatest benefit of PCSK9i on MACE risk among patients with recent acute coronary syndrome were those with optimal LDL-C levels and mildly elevated Lp(a) [43]. Taking into account that similar findings were obtained in the FOURIER study [44], Lp(a) could be a reliable biomarker of residual risk, although certain confounding effects remain due to its potential role in atherothrombosis. Of note, Lp(a) is not a routine lipid parameter, and its assessment could be difficult due to heterogeneity of apo(a) isoforms. At present, there is no standardized assay for the measurement of either Lp(a) plasma level or apo(a) size isoforms [45]. However, Lp(a) is a unique lipid biomarker whose plasma concentration is predominantly determined by variations of a single gene and, therefore, could be measured once in a lifetime [20].

## 4. Atherogenic Lipoproteins and Residual Cardiovascular Risk

**Figure 1:**
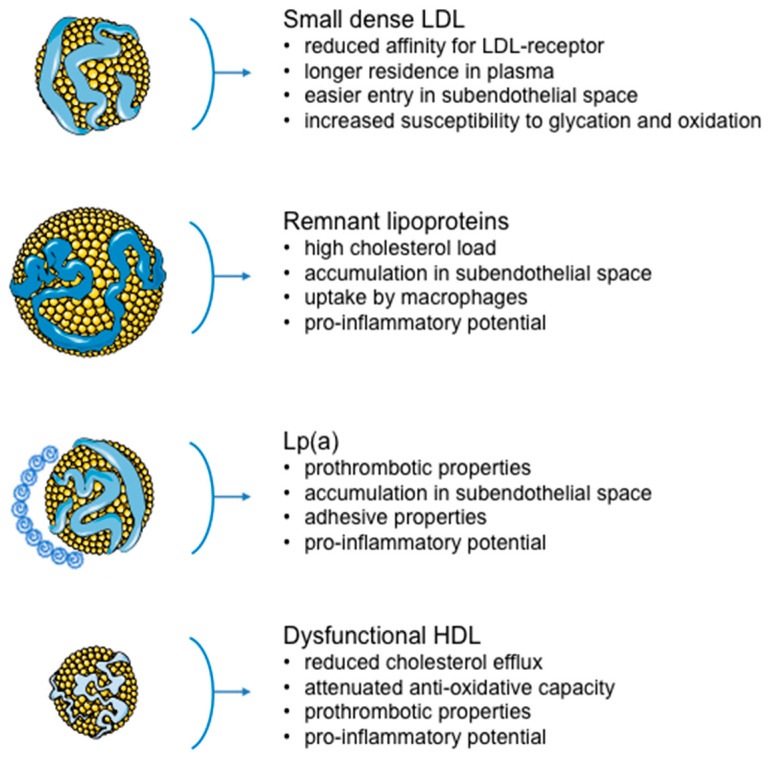
The contribution of atherogenic lipoproteins to residual risk.

Overall, the data discussed here suggest a clear benefit of targeting atherogenic lipoproteins for further reduction of residual risk (Figure 1), which is of particular clinical relevance for CVD patients with other co-morbidities, such as diabetes. For instance, the results of a recent study confirmed a positive impact of aggressive lipid-lowering therapy on each of the above-mentioned lipid biomarkers in patients with diabetes, regardless of the presence of atherogenic dyslipidemia [46]. On the other hand, the usefulness of specific HDL-based biomarkers for CVD risk estimation is debatable. Although the inverse association between HDL-C and cardiovascular morbidity and mortality was recorded as early as the second half of the 20th century [47], the decades-long journey of HDL research did not end with univocal results. 

Recent findings of the CANHEART Study [48] have demonstrated that, regardless of a consistent link with overall mortality and morbidity, HDL-C cannot be qualified as a specific cardiovascular risk factor. Yet, today it is widely accepted that HDL is much more than its cholesterol content. Even if reverse cholesterol transport is a principal aspect of HDL synthesis, metabolism and their biological role, the complexity of these particles suggests their broader functional properties. Consequently, the possible contribution of this lipoprotein to the impairment of cardiovascular homeostasis should not be considered solely through HDL-C concentration. Appreciating all these facts, contemporary science considers alterations of HDL protein–lipid composition and subsequent loss of its functionality as important contributors to atherosclerosis (Figure 1). 

## 5. Dysfunctional HDL

The term “dysfunctional HDL” has been forged with an aim to illustrate the incapability of HDL particles to perform protective, anti-atherosclerotic activities [49]. It has been firmly confirmed that an altered vascular environment, i.e., pro-inflammatory and pro-oxidative milieu, can provoke structural changes of HDL and diminish its functionality [49]. Key structural contributors and thereby markers of HDL functional capacity, including apolipoprotein A-I, myeloperoxidase and paraoxonase 1, are well known. Currently, sphingosine-1-phosphate (S1P), a lipid moiety associated with HDL, is widely investigated regarding the functional properties of this lipoprotein. 

Previous studies demonstrated that S1P exhibits atheroprotective effects by regulation of survival, proliferation, differentiation, and mobility of different types of cells, which participate in maintaining vascular homeostasis [50,51]. S1P is dominantly transported in a complex with apolipoprotein M, which is a protein constituent of HDL, and it has been demonstrated that biological functions of both HDL and S1P are dependent on their mutual associations [51]. In a recent longitudinal study, Soria-Florido et al. [52] have reported a significant inverse association between S1P levels in apolipoprotein B-depleted plasma and the onset of myocardial infarction, thus confirming the contribution of S1P to the atheroprotective capability of HDL. Furthermore, it has been shown that the S1P enrichment of dysfunctional HDL can contribute to the restoration of its vasoprotective function [53], which suggests the role of S1P in maintaining HDL functionality. 

Besides lipid species, numerous protein constituents of HDL are responsible for its antioxidative, anti-inflammatory, anti-apoptotic and endothelium-protective effects [54]. In addition, it was shown that small non-coding RNA, specifically micro-RNA (miRNA), is also transported and delivered to target cells by HDL and that HDL-miRNA composition differs in healthy and hypercholesterolemic subjects [55]. Recent findings of Ben-Aicha et al. [56] obtained on animal models suggest that hypercholesterolemia can induce alterations of HDL-miRNA content with consequent changes in the expression of the target gene. These findings extend previous understanding of HDL functionality and add another potential HDL-related effect: changes to gene expression. 

One interesting constraint should be noted with respect to the proposed antiatherogenic characteristics of HDL and its capacity as a biomarker. As recently summarized by Beazer et al. [57], given the fact that extracellular vesicles (EC) are frequently co-isolated with HDL, it is possible that functional properties of HDL could at least partly be attributed to biologically active EC. Thus, the question of dysfunctional HDL and its contribution to residual cardiovascular risk should also be evaluated considering the possible influence of EC. In recent years, several additional mechanisms have been proposed to improve the understanding of HDL dysfunctionality, paving the way for novel HDL-related biomarkers of residual risks, such as cholesterol efflux capacity [58] and HDL-inflammatory index [59].

Finally, it should be noted that, in parallel with futile attempts to decisively confirm the independent contribution of low HDL-C to CVD development, a plethora of evidence suggest modulatory roles of HDL in non-cardiovascular diseases, such as diabetes, disorders of the central nervous system, infections, kidney diseases or cancer [60,61,62,63]. Thus, HDL composition and functionality should be observed and evaluated in a broader sense as biomarkers of metabolic alterations associated with a range of pathological conditions.

## 6. The Role of Innovative Antidiabetic Therapies on Atherogenic Lipoproteins 

There is increasing interest in the effects of novel antidiabetic treatments on atherogenic lipoproteins since such therapies significantly impact the cardiovascular outcome in patients with type-2 diabetes; sodium-glucose cotransporter-2 inhibitors (SGLT-2i) and glucagon-like peptide-1 receptor agonists (GLP-1RAs) have been shown over the last few years to reduce cardiovascular events and mortality, while dipeptidyl peptidase-4 inhibitors (DPP-4i) have a neutral effect with cardiovascular safety, although with no benefit [64,65]. The mechanisms involved in such divergent cardiovascular effects are still largely unclear, and it has been postulated that this may be linked to a direct anti-atherosclerotic action possessed by some of these novel agents [66,67]. It is therefore of importance to assess to which extent these innovative antidiabetic treatments may modulate atherogenic lipoproteins. 

Agents in both classes of SGLT-2i and GLP1-RAs, including dapagliflozin, exenatide and liraglutide, have shown the ability to significantly reduce levels of small, dense LDL in patients with type-2 diabetes [68,69,70,71]. This is consistent with previous findings from different meta-analyses investigating the impact of innovative antidiabetic therapies on plasma lipids [72,73], which have shown that the main effect of both SGLT-2i and GLP1-RAs was in lowering plasma triglyceride levels and therefore suggesting a major role of these agents in reducing triglyceride-rich lipoproteins, the direct precursors of small, dense LDL [74]. By contrast, the DPP-4i sitagliptin did not reduce small dense LDL concentrations [68], very similar to what was previously obtained with the use of traditional antidiabetic agents, such as rosiglitazone [75], who do not has a beneficial cardiovascular outcome [76]. 

All the above suggest a divergent effect of novel antidiabetic therapies on atherogenic small, dense LDL: SGLT-2i and GLP1-RAs reduce their levels, while DPP-4i do not. This is fully in line with the divergent effect of such therapies on the cardiovascular outcome, as previously discussed. Of interest, some of the agents able to reduce small dense LDL, such as exenatide and liraglutide, are also concomitantly reducing the concentrations of the pro-atherogenic adipokine resistin [77,78]; since previous studies have shown that small, dense LDL and resistin are closely linked for increased risk of cardiovascular diseases [79], we cannot exclude this pathway may represent a valuable mechanistic explanation for the cardiovascular benefit of most, but not all, antidiabetic therapies.

However, it is somewhat puzzling that findings on Lp(a) are totally oppositive: agents in both classes of SGLT-2i and GLP1-RAs, such as empagliflozin and liraglutide, have shown no significant effect [80,81] while the DPP-4i linagliptin and gemigliptin significantly reduced Lp(a) concentrations [82]. This has an important clinical significance since recent studies have highlighted the role of Lp(a) as a marker of residual risk in patients with diabetes and established cardiovascular diseases [83]. Finally, oral semaglutide, the latest novel antidiabetic agent introduced in the market, has been recently shown to significantly improve fasting and postprandial glucose and lipid metabolism [84]; notably, these beneficial effects were quite similar to those previously reported with the use of the once-weekly subcutaneous formulation of semaglutide [85].

## 7. Conclusions

The presence of residual cardiovascular risk is a current dilemma in clinical practice; indeed, despite optimal management and treatment, a considerable proportion of patients still undergo major cardiovascular events. This emphasizes the need for further research in the field in order to clearly assess the effects of innovative treatments on different novel biomarkers, including atherogenic lipoproteins, such as small dense LDL, Lp(a) and dysfunctional HDL. This is particularly important during the current coronavirus COVID-19 pandemic since a higher number of cardiovascular diseases, such as acute myocardial infarction, have been reported [86]. Insights and learnings from recent experience can help us to guide future management [87], offering to our patients the best available options for proper reduction of cardiovascular risk.
